# Supplementary material for: Air pollution exposure, respiratory consequences, and perceptions among urban African children living in poor conditions – A case study in Abidjan, Côte d’Ivoire
Source: PLOS Glob Public Health. 2025 Apr 29;5(4):e0003703. doi: 10.1371/journal.pgph.0003703 (PMC12040247; doi:10.1371/journal.pgph.0003703)
Supplement: Attachment — Submitted filename: Response to Reviewers.docx [file pgph.0003703.s003.docx]

Revision of the article: “Air pollution exposure, respiratory consequences, and perceptions among urban African children living in poor conditions – a case study in Abidjan, Côte d'Ivoire”

Author’s response to reviewer’s comments

| **Reviewer comments** | **Authors’ response** |
| --- | --- |
| **Reviewer 1** |  |
| Reviewer’s report | We thank Reviewer 1 for the interest and time accorded to the revision of our article. We addressed their questions and comments in a point-by-point response below.  In addition, we have taken this opportunity for minor edits to improve the writing that are in track change in the manuscript. We hope that this has also contributed to improve the overall quality of our manuscript. |
| Line 109: the study time was stipulated, but I think the different tests would have been conducted in different seasons in that area. This will enable accurate comparisons of the results. Equally, the study that was 2016 did not take into consideration the various seasons of the area | We thank the reviewer for their comment on the important aspect of seasonality, indeed an important factor to consider. Data collection was conducted in full during rainy season. The 2016 (Kouao) study data were collected from February to August, which corresponds to an overlap of several seasons and our data may not be fully comparable for that reason.  Indeed, particulate matter (PM) concentrations are lower during the rainy season compared to the dry season, especially outdoors, mainly due to the Harmattan, a wind coming from the Sahara that carries desert dust during dry season, thereby contributing to increased PM levels. Additionally, the dry season promotes the prolonged suspension of particles in the air, while during the rainy season, these particles are partially removed through atmospheric washing processes (1).  We have now mentioned the season in the manuscript, in the Study design section, line 122 as follows:  *“The SEPol-CI study was a multidisciplinary cross-sectional study with enrollments conducted between June 23 and August 25, 2022, during the rainy season. ”*  In light of your comment, it seemed important to mention also the lack of consideration of seasonality in the study's limitations. Therefore, we have added the following sentence, line 513 :  “It is crucial to highlight the impact of seasonality on pollution and respiratory symptoms, particularly regarding the dry season, wet season, and the presence of Harmattan. In both CHAIRPOL and this study, these factors were not considered, and it will be essential to integrate them into future projects.”  1. https://doi.org/10.4209/aaqr.2020.05.0201 |
| Line 115: I don’t think children can respond in-depth to an interview. We can say interviewing children aged 5-10 | We agree with the reviewer on the fact that interviews with children are not in-depth. We therefore removed *“in-depth”* in the revised version of the manuscript, line 128. |
| Line 154: you had a range of age done, say 5 and over, but remain within the range that was specified. | We indeed included only children aged 5 to 10 years. Spirometry measurements were performed on all the children. However, for those unable to perform the forced expirations required for this test, we conducted Rint measurements instead. This is a simpler and non-invasive examination that requires minimal cooperation. In practice, it turned out that the youngest children underwent Rint measurements instead of spirometry.For clarity, here is the modification at line 172:  *" Respiratory function tests were carried out by a medical doctor, spirometry measurements were taken in children, and airway interruption resistance (Rint) were performed on children who were unable to perform spirometric curves".* |
| Line 182: correct the grammar in that sentence Remark: When abbreviating words for the first time, define them | We corrected the grammar in the sentence line 201 that now reads as follows:  *“In children unable to undergo a full spirometry, OVD was defined as a Rint > 150% of the theoretical value with reversibility of obstruction objectified by a decrease in Rint ≥ 35% of the predicted value after reversibility testing.”* |
| Line 214: correct the phrase home fish smoking fish | We have addressed this syntax error in the revised version of the manuscript line 233 that now reads :  *"fish smoking activity in the household."*  For consistency, the wording of this sentence has also been modified in the results section, line 259 :  “*29 households reported practicing fish smoking activity ”* |
| Line 221: how many interviews were conducted ? Specify it | We conducted 8 semi-structured interviews with adults included 5 women and 3 men.  We have added the following statement in the results section line 319:  *"We conducted semi-structured interviews with 5 women and 3 men"* |
| Line 259: remove one ‘’had’’ | We have corrected the typo. |
| Line 339: you should be able to specify the number of people who raised the need for public awareness of pollution to be addressed | Since this is a qualitative analysis based on semi-structured interviews, it is not recommended to specify the number of people who mentioned a particular idea. The focus should be on the concept raised rather than the number. Therefore, we removed the term "Several" to eliminate any implication of quantification and the sentence now reads, line 365:  *“Respondents suggested raising public awareness to start addressing the air pollution problem*”. |
| Line 356: remove one ‘’if they did’’ | We have corrected the typo. |
| Remark: From lines 334 to 358, most of your observations came from interview 3. Why? | This particular interview was notably clear, rich, and comprehensive in terms of the points raised about air pollution. The quality of the interviews varied depending on the interviewee. Some interviewees delved deeply into only certain aspects of the semi-structured questionnaire, while others expanded on each of the topics addressed.  However, due to the need to prioritize and keep the manuscript concise, particularly in relation to the quantitative section, we chose not to further extend the qualitative part. |
| Personal views: The work is quite rich and educative. I think the sample size can be increased and different seasons considered. It will improve the results of the next studies. There was no similarity between youngsters residing in gas-powered homes and those whose primary fuel source was coal or firewood. The Chai square was not adequately used to compare the variables (the Chi-Square test is used to determine whether there is a significant association between categorical variables). and you mentioned it in your methods. | We thank the reviewer for their appreciative comments, and the suggestion to increase sample size and consider different seasons which are valuable for this manuscript and will be used for future projects created on the same topic.  Regarding statistical analyses, we performed the following tests: Chi-square test of independence or Fisher's exact test for categorical variables, and Wilcoxon-Mann-Whitney test for ordinal or continuous variables. We used Chi-square tests, Fisher's exact tests, Wilcoxon tests, and Kruskal-Wallis tests to select independent variables with a P value <0.20, which were then included in a logistic regression model. The results of the logistic regression are not presented in this article due to non-significant findings. For clarity, we mentioned them in the results section with the following sentence line (304):  “*Logistic regressions assessing factors associated with the presence of LFI in children were not statistically significant (results not presented in the article)*.” |
| **Reviewer 2** |  |
| The strength of this study lies in using mixed method approach and also of involving both parents and children to understand their perception of air pollution and it's effects. However a small sample size and fewer examinations of spirometry and clinical assessments could be the reason for not finding any significant association with the risk factors. | We thank Reviewer 2 for their thorough review of our manuscript our manuscript and their comments. We have answered questions and comments in a point-by-point response in the table below.  This study was indeed a pilot study with a small sample size, which may have led to negative findings. We have added this point among the limitations on line 517.  *“As our sample size is small (65 households and 124 children), we cannot generalize our results to the entire area but rather consider this study as a case study, hence the fact that it is a pilot study. The sample size could be the reason for not finding any significant association with the risk factors. »* |
| Provide Reference to this statement in introduction "In 2019, 99% of the world's population lived in areas where air pollutant concentration exceeds WHO recommended air quality thresholds." | This statement comes from the WHO report that was updated on September 13, 2024, regarding "Ambient (Outdoor) Air Pollution. Consequently, we have updated the bibliography. |
| Line 79: "high" burden | We have corrected the typo. |
| Line 104: "improve" | We have corrected the typo. |
| Line 172: respiratory symptom or asthma symptom not clear | In our study, we focused on the frequency of asthma like respiratory symptoms derived from the ISAAC questionnaire: presence of wheezing, history of asthma attack, sleep disturbed by wheezing, speech limited, wheezing in the chest during or after exercise, and dry cough at night in the last 12 months. However, we also considered other respiratory symptoms such as daily cough or throat irritation. We have revised the manuscript for greater clarity with the following updated version.  In the section: Main respiratory health outcomes and definitions, line 188.  *“We considered as respiratory symptoms suggestive of asthma defined by the ISAAC questionnaire: presence of wheezing, history of asthma attack, sleep disturbed by weezing, speech limited, wheezing in the chest during or after exercise, dry cough at night, in the last 12 months. Pulmonary function assessments were performed using spirometry and Rint, measured with a Spirodyn pneumotachograph. »*  And in the section: Other health outcomes, line 209:  *“Children's health data included: clinical signs on auscultation by a physician, respiratory symptoms (daily cough, throat irritation) and history, vital parameters, spirometric parameters, and Rint measurements.”* |
| Line 181: unable to "undergo" | We have corrected the grammar |
| Provide any references or justification for the cut-offs chosen for OVD, RVD and asthma. | We have defined asthma on spirometry using the Global Initiative for Asthma (GINA) definition as reversibility of FEV1 >12% compared to the pre-bronchodilator FEV1 value (2) in the presence of an obstructive syndrome which is usually defined by FEV1/FVC post-bronchodilator < 70% (2). In children, for better accuracy, an adjusted value is used based on age, sex, height, and ethnicity: the 5th percentile of reference values (called the Lower Limit of Normal - LLN) (2). However, where children have difficulty blowing for more than 3 seconds into the mouthpiece during spirometry tests, the Tiffeneau ratio may be normal or nearly normal. Moreover, FEV1 and FVC values may be abnormally low. Peak expiratory flow (PEF) and maximum inspiratory flow rates can assist in diagnosing extra-thoracic airway obstructions (3).  The definitions retained for this study were:  Normal Spirometry: defined by a forced vital capacity (FVC) ≥ 80%, a forced expiratory volume in the first second (FEV1) ≥ 80%, a Tiffeneau ratio (FEV1/FVC) ≥ 80%, and a peak expiratory flow (PEF25-75) ≥ 70% of the theoretical value for each child calculated based on their weight and height.  Obstructive Syndrome: characterized by a decrease in the FEV1/FVC ratio ≤ 80% or a PEF25-75 < 70% of the theoretical value and/or an FEV1 < 80% of the theoretical value. Diagnosis: The obstructive pulmonary syndrome can be reversible (improvement with bronchodilator treatment), defined as asthma in spirometry, or fixed. The criterion for reversibility is an increase in the maximum expired volume in 1 second (FEV1) or forced vital capacity (FVC) of 12% from baseline.  Restrictive Syndrome (or restrictive ventilatory disorder; RVD): defined as a decrease in mobilizable lung volumes. Diagnosis: FVC < 80% and a PEF 25-75 or FEV1 normal, respectively ≥ 70% and ≥ 80%.  Mixed Syndrome (Mixed ventilatory disorder): combination of obstructive and restrictive ventilatory disorders, characterized by an FVC < 80%, a PEF 25-75 < 70%, and/or an FEV1 < 80%.  These definitions have been presented in the manuscript line 194 :  *“A non-reversible OVD was defined by a forced expiratory flow at 25-75% of forced vital capacity (FEF25-75) < 70% of the theoretical value and/or forced expiratory volume in one second (FEV1) < 80% of the theoretical value and non-reversible on the bronchodilator reversibility test. A RVD was defined by a forced vital capacity (FVC) < 80% and normal FEF25-75 or FEV1, respectively ≥70 % et ≥ 80%. A mixed disorder was characterized by FVC < 80%, FEF25-75 < 70% and or FEV1 < 80%. Asthma was defined by a reversible OVD with a FEV1 greater than 12% of baseline on post-test reversibility to bronchodilatator. In children unable to undergo a full spirometry, OVD on Rint was defined as a Rint > 150% of the theoretical value with reversibility of obstruction objectified by a decrease in Rint ≥ 35% of the predicted value after reversibility testing.”*  2. https://ginasthma.org/2023-gina-main-report/  3. https://splf.fr/wp-content/uploads/2014/07/RMR2006_pftERS.pdf |
| Line 214: Tobacco | We have corrected the typo. |
| Line 214: what is "home fish smoking fish"? | As also noted by the first reviewer, wehave addressed this syntax error in the new version of the manuscript by replacing *“home fish smoking fish”* with *"fish smoking activity in the household."* line 233 |
| Was the amount of time the child spends at home, presence of child during cooking activities, ventilation inside home assessed? These parameters could be important factors that need to be accounted for. | We agree that a detailed assessment of time spent at home and the child’s presence when cooking as well as a thorough assessment of ventilation for each household would be interesting to investigate and to take into consideration in a large study. However in this pilot study we assessed those factors with.  Regarding the presence of the child during cooking activities, we simply asked mothers about the possible presence of the child near the stove during cooking which we did not report initially, suspecting a possible prevarication bias.  We have add this variable to the descriptive data, Table 1:*“Child near the stove during cooking times.”*  Which shows that 46 mothers (71%) reported that their children were never near the fire during cooking times, 11 (17%) sometimes near, 7 (11%) often, and 1 (1%) very often near the cooking fire.  We did not assess ventilation in the home but assess the type of kitchen for each household as being: i) closed, with window/screen, ii) closed, windowless, iii) unenclosed, in courtyard or corridor. |
| Table 1: Explain what is hevea? In table it is Hevea in the result section you mention rubberwood. | Hevea is the rubber tree. Women use pieces of white rubber filaments recovered from the rubber tree, which are dried and used to ignite their biomass combustion fire. We have replaced and standardized all instances of the terms *"hevea"* and *"rubberwood"* with *"dry rubber"* line 276 and Table 1 . |
| Type of cooking equipment: Explain the difference between traditional oven, improved cooker and gas cooker. | The variables *“Type of fuel”* and *“Type of cooking* equipment” are two distinct variables.  - “Type of fuel” refers to the type of combustible used for cooking (gas, wood, and coal)  - “Type of cooking equipment” refers to the cooking materials (traditional oven, improved cooker, or gas cooker).  To provide more clarity, as you pointed out, we have added definitions for the types of stoves used by women line 272 : “*In this study, the improved stove is distinguished from the traditional one by its combustion chamber, which limits smoke propagation, reduces cooking time, and decreases the amount of fuel used. The gas cooker is a butane gas cylinder with a pot support.”* |
| Has stove type and fuel both been mixed in this variable ? | For the analyses, the variable “type of cooking” was tested as is, but we also recoded this variable into a binary variable by combining the categories “gas and coal” and “gas, coal, and wood.” This resulted in a binary variable: Gas only/mix of gas and biomass (coal or wood). |
| Table 2 : replace size with height | We have corrected the typo. |
| Line 433: Incomplete sentence, state the numbers “The prevalence of wheezing in the chest during the year, a dry cough at night.” | Indeed, it is an error, this sentence should be removed as it relates to the previous sentence line 465 :  *“Of the 124 children included, the prevalence of respiratory symptoms suggestive of asthma was high.”*  To harmonize the terms throughout the article, wereplaced in this same line, line 465:  *“bronchial symptoms suggestive of asthma”* by *“respiratory symptoms suggestive of asthma”* |
| Line 436: “These results are far superior to other studies”. Which results are you speaking about? The prevalence in your study for wheezing is much higher than in the Camerron study or Djibouti study. You can state “The prevalence is much higher in our study compared to other regions” | We thank the reviewer for their suggestion and have rephrased the sentence as suggested in the revised version of the manuscript. |
| Line 441: “Because of the difficulty some children have in making spirometry curves correctly, and because of the variability of spirometry curves at these ages, it would be preferable not to take into account spirometric data exclusively.” Instead state “Due to the difficulty some children have in correctly performing spirometry and because of the variability of spirometry curves at these ages, it would be preferable not to take into account spirometric data exclusively.” | We thank the reviewer for their suggestion and have rephrased the sentence as suggested in the revised version of the manuscript. |
| Please mention the correct sample size Line 477: As our sample size is small (59 households and 117 children), Line 231: We included 124 children from 65 households | Indeed, my sample consists of 124 children from 65 households; we have corrected this error in the revised version of the manuscript. |
| **Reviewer 3** |  |
| I found the article engaging, particularly the qualitative section that explored perceptions of air pollution. The insights shared by the children regarding the impacts of air pollution were especially valuable. Here are my specific comments: | We thank Reviewer 3 for the interest and time accorded to the revision of our article. We addressed their questions and comments in a point-by-point response below.  In addition, we have taken this opportunity for minor edits to improve the writing that are in track change in the manuscript. We hope that this has also contributed to improve the overall quality of our manuscript. |
| INTRODUCTION  Line 73: In 2016, approximately 543,000 deaths of children under 5 and 52,000 deaths of children aged 5 to 15 were attributed to household and outdoor air pollution. Could be updated to - In 2021, more than 700,000 deaths in children under 5 years of age were from diseases linked to air pollution in 2021. Of this, over 500,000 deaths were linked to exposure to household air pollution. (IHME, Global burden of disease 2024) | We were able to update this data thanks to the “State of Global Air from 2024.” The new data included in the latest version of the manuscript is as follows line 75:  *“In 2021, more than 700,000 deaths in children under 5 years of age were from diseases linked to air pollution.”* |
| Line 88: In Côte d'Ivoire, outdoor air pollution is mainly due to anthropogenic activities such as burning of dumped waste in open air, fish smoking activities.. Complete this sentence. What other anthropogenic activities are being referred to? | The anthropogenic activities related to air pollution in Côte d’Ivoire are: burning of dumped waste in open air, the use of biomass for domestic cooking and fish smoking activities, unregulated industries, the use of outdated vehicles, poor quality of the roads, and biomass fuel for cooking.  We have added the various pollution sources in the corresponding paragraph along with the associated references line [97-109]. |
| Line 103: This interdisciplinary work could help to understand pollution phenomena and find concrete solutions to protect exposed populations and improved their quality of life. Rephrase this sentence. What is meant by pollution phenomena? | By *“pollution phenomena”* we meant the issue of air pollution, its sources, and the consequences associated with this pollution.We agree that "phenomena" is a general term. The new proposed sentence is as follows, line 115 :  *“This interdisciplinary work could contribute to a better understanding of air pollution issues and find concrete solutions to protect exposed populations and improve their quality of life.”* |
| METHODS  DATA ANALYSIS Quantitative data analysis Line 214: ‘home fish smoking fish’ A bit confusing! Rephrase as ‘smoking fish/ meat in the house/ courtyard | As stated in responses to reviewer 1 and 2, we have addressed this syntax error in the revised version of the manuscript line 233 that now reads :  *"fish smoking activity in the household."*  For consistency, the wording of this sentence has also been modified in the results section, which now reads in the revised version of the manuscript line *259 :*  “*29 households reported practicing fish smoking activity ”* |
| Line 264: Table 2. Demographic and medical characteristics, clinical signs, vital parameters and prevalence of lung function impairment of children Clinicals signs reported on the day of clinical examination | We thank the reviewer for pointing out this error. We have removed *"nation"* in the revised version of the manuscript that should not have been included in Table 2. |
| DISCUSSION  Line 495: Respiratory fonction test results confirmed the presence of ventilatory disorders and asthma in these children. Correct ‘fonction’ to ‘function’ | We have corrected the typo. |
