# Supplementary material for: Air pollution exposure, respiratory consequences, and perceptions among urban African children living in poor conditions – A case study in Abidjan, Côte d’Ivoire
Source: PLOS Glob Public Health. 2025 Apr 29;5(4):e0003703. doi: 10.1371/journal.pgph.0003703 (PMC12040247; doi:10.1371/journal.pgph.0003703)
Supplement: Attachment — Submitted filename: Reviewers report for PGPH-D-24-01910.docx [file pgph.0003703.s002.docx]

Reviewer’s report

Line 109: the study time was stipulated, but I think the different tests would have been conducted in different seasons in that area. This will enable accurate comparisons of the results. Equally, the study that was 2016 did not take into consideration the various seasons of the area

Line 115: I don’t think children can respond in-depth to an interview. We can say interviewing children aged 5-10

Line 154: you had a range of age done say 5 and over, but remain within the range that was specified.

Line 182: correct the grammar in that sentence

Remark: When abbreviating words for the first time, define them

Line 214: correct the phrase home fish smoking fish

Line 221: how many interviews were conducted specify it

Line 259: remove one ‘’had’’

Line 339: you should be able to specify the number of people who raised the need for public awareness of pollution to be addressed

Line 356: remove one ‘’if they did’’

Remark: From lines 334 to 358 most of your observations came from interview 3, why?

Personal views:

The work is quite rich and educative. I think the sample size can be increased and different seasons considered, It will better the results of the next studies.

There was no similarity between youngsters residing in gas-powered homes and those whose primary fuel source was coal or firewood.

The Chai square was not adequately used to compare the variables (The Chi-Square test is used to determine whether there is a significant association between categorical variables). and, you mentioned it in your methods.
